# Bis(2-bromo-5-methyl­phen­oxy)methane

**DOI:** 10.1107/S1600536811034805

**Published:** 2011-08-31

**Authors:** Jun-long Niu, Xia Wang, Lin-bao Zhang, Mao-ping Song

**Affiliations:** a Department of Chemistry, Henan Key Laboratory of Chemical Biology and Organic Chemistry, Zhengzhou University, Zhengzhou 450052, People’s Republic of China; b Pharmacy College, Henan University of Traditional Chinese Medicine, Zhengzhou 450008, People’s Republic of China

## Abstract

The complete mol­ecule of the title compund, C_15_H_14_Br_2_O_2_, is generated by the application of crystallographic twofold symmetry, with the central C atom lying on the rotation axis. The dihedral angle between the benzene rings is 62.4 (3)°. In the crystal, short Br⋯Br contacts [3.4885 (16) Å] occur.

## Related literature

For background to bromo­aromatic compounds, see: Butler & Walker (1993 ▶); Seevers & Counsell (1982 ▶). For a related structure, see: Zheng *et al.* (2004 ▶).
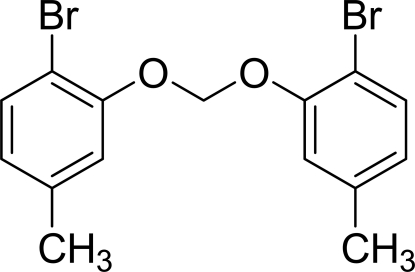


## Experimental

### 

#### Crystal data

C_15_H_14_Br_2_O_2_
*M* *_r_* = 386.08
Orthorhombic, 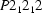

*a* = 10.7752 (11) Å
*b* = 15.8690 (17) Å
*c* = 4.3272 (10) Å
*V* = 739.9 (2) Å^3^
*Z* = 2
Cu *K*α radiation
μ = 6.91 mm^−1^
*T* = 291 K
0.35 × 0.30 × 0.30 mm

#### Data collection

Agilent Xcalibur Eos Gemini diffractometer
Absorption correction: multi-scan (*CrysAlis PRO*; Agilent, 2011 ▶) *T*_min_ = 0.196, *T*_max_ = 0.231
1505 measured reflections
1022 independent reflections
754 reflections with *I* > 2σ(*I*)
*R*_int_ = 0.044

#### Refinement

*R*[*F* ^2^ > 2σ(*F* ^2^)] = 0.055
*wR*(*F* ^2^) = 0.123
*S* = 1.02
1022 reflections
88 parameters
H-atom parameters constrained
Δρ_max_ = 0.36 e Å^−3^
Δρ_min_ = −0.41 e Å^−3^
Absolute structure: Flack (1983 ▶), 212 Friedel pairs
Flack parameter: −0.11 (9)

### 

Data collection: *CrysAlis PRO* (Agilent, 2011 ▶); cell refinement: *CrysAlis PRO*; data reduction: *CrysAlis PRO*; program(s) used to solve structure: *SHELXS97* (Sheldrick, 2008 ▶); program(s) used to refine structure: *SHELXL97* (Sheldrick, 2008 ▶); molecular graphics: *OLEX2* (Dolomanov *et al.*, 2009 ▶); software used to prepare material for publication: *SHELXL97*.

## Supplementary Material

Crystal structure: contains datablock(s) global, I. DOI: 10.1107/S1600536811034805/hb6383sup1.cif

Structure factors: contains datablock(s) I. DOI: 10.1107/S1600536811034805/hb6383Isup2.hkl

Supplementary material file. DOI: 10.1107/S1600536811034805/hb6383Isup3.cml

Additional supplementary materials:  crystallographic information; 3D view; checkCIF report

## Figures and Tables

**Fig. 1.**
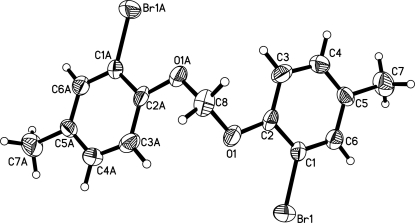
View of the title compound, showing 30% probability ellipsolids. Atoms with suffix A are generated by (1–x, 2–y, z0.

**Fig. 2.**
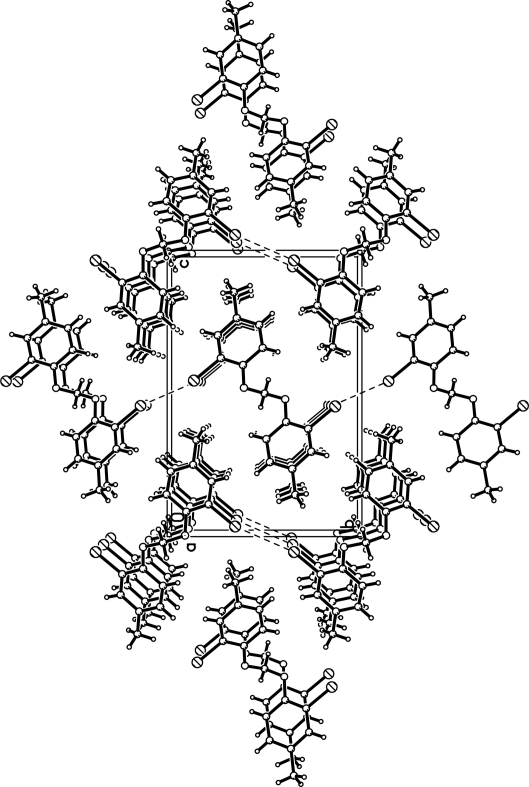
A view of the crystal packing along the c axis, with short Br···Br contacts indicated by dashed lines.

## supplementary crystallographic information

### Comment

Bromoaromatic compounds have proven to be an important class of molecules in synthetic organic chemistry. They have been used as key intermediates in the preparation of oganometallic reagents and play vital roles in transition metal mediated coupling reactions (Butler *et al.*, 1993; Seevers *et al.*, 1982). In this paper, we synthesized the title compound and reported its crystal structure here. The title compound was synthesized by the reaction of 2-bromo-4-methylphenol, dibromomethane with potassium carbonate. The C—C—C angles within the aromatic moiety cover a range 117.7 (8) - 122.5 (8) °, and the two benzene rings make a dihedral angle of 62.5° (Fig. 1). The O and Br atoms are essentially coplanar with the benzene ring to which they are attached, with the deviation of 0.0074 Å. In addition, the benzene rings between the adjacent molecules are stacked in a face-to-face orientation with the distance of 3.701 Å, a distance longer than the π–π stacking distances of 3.33 - 3.53 Å reported elsewhere (Zheng *et al.*, 2004), indicating no π–π stacking is observed for this compound.

### Experimental

A mixture of 2-bromo-4-methylphenol (188 mg, 1 mmol), potassium carbonate (691 mg, 5 mmol) and dibromomethane (0.75 mmol) in acetone (5 ml) was heated to reflux for 12 h. The product was isolated and recrystallized from dicholomethane/hexane, colorless prisms of the title compound were obtained.

### Refinement

H atoms were generated geometrically and refined as riding atoms with C-H = 0.93Å and Uiso(H) = 1.2 times Ueq(C).

### Crystal data

**Table:**

C_15_H_14_Br_2_O_2_	*D*_x_ = 1.733 Mg m^−^^3^
*M**_r_* = 386.08	Cu *K*α radiation, λ = 1.54178 Å
Orthorhombic, *P*2_1_2_1_2	Cell parameters from 443 reflections
*a* = 10.7752 (11) Å	θ = 4.1–69.9°
*b* = 15.8690 (17) Å	µ = 6.91 mm^−^^1^
*c* = 4.3272 (10) Å	*T* = 291 K
*V* = 739.9 (2) Å^3^	Prism, colorless
*Z* = 2	0.35 × 0.30 × 0.30 mm
*F*(000) = 380	

### Data collection

**Table:**

Agilent Xcalibur Eos Gemini diffractometer	1022 independent reflections
Radiation source: fine-focus sealed tube	754 reflections with *I* > 2σ(*I*)
graphite	*R*_int_ = 0.044
Detector resolution: 16.2312 pixels mm^-1^	θ_max_ = 66.9°, θ_min_ = 5.0°
ω scans	*h* = −12→9
Absorption correction: multi-scan (*CrysAlis PRO*; Agilent, 2011)	*k* = −18→14
*T*_min_ = 0.196, *T*_max_ = 0.231	*l* = −3→4
1505 measured reflections	

### Refinement

**Table:**

Refinement on *F*^2^	Secondary atom site location: difference Fourier map
Least-squares matrix: full	Hydrogen site location: inferred from neighbouring sites
*R*[*F*^2^ > 2σ(*F*^2^)] = 0.055	H-atom parameters constrained
*wR*(*F*^2^) = 0.123	*w* = 1/[σ^2^(*F*_o_^2^) + (0.040*P*)^2^] where *P* = (*F*_o_^2^ + 2*F*_c_^2^)/3
*S* = 1.02	(Δ/σ)_max_ < 0.001
1022 reflections	Δρ_max_ = 0.36 e Å^−^^3^
88 parameters	Δρ_min_ = −0.41 e Å^−^^3^
0 restraints	Absolute structure: Flack (1983), 212 Friedel pairs
Primary atom site location: structure-invariant direct methods	Flack parameter: −0.11 (9)

### Special details

**Table:**

Geometry. All e.s.d.'s (except the e.s.d. in the dihedral angle between two l.s. planes) are estimated using the full covariance matrix. The cell e.s.d.'s are taken into account individually in the estimation of e.s.d.'s in distances, angles and torsion angles; correlations between e.s.d.'s in cell parameters are only used when they are defined by crystal symmetry. An approximate (isotropic) treatment of cell e.s.d.'s is used for estimating e.s.d.'s involving l.s. planes.
Refinement. Refinement of *F*^2^ against ALL reflections. The weighted *R*-factor *wR* and goodness of fit *S* are based on *F*^2^, conventional *R*-factors *R* are based on *F*, with *F* set to zero for negative *F*^2^. The threshold expression of *F*^2^ > σ(*F*^2^) is used only for calculating *R*-factors(gt) *etc*. and is not relevant to the choice of reflections for refinement. *R*-factors based on *F*^2^ are statistically about twice as large as those based on *F*, and *R*- factors based on ALL data will be even larger.

### Fractional atomic coordinates and isotropic or equivalent isotropic displacement parameters (Å^2^)

**Table:**

	*x*	*y*	*z*	*U*_iso_*/*U*_eq_	Occ. (<1)
Br1	0.85154 (9)	0.95619 (6)	0.6233 (4)	0.0946 (6)	
O1	0.6042 (5)	0.9803 (3)	0.3521 (17)	0.0668 (18)	
C1	0.7175 (7)	0.8802 (4)	0.624 (3)	0.054 (2)	
C2	0.6083 (7)	0.9029 (5)	0.489 (2)	0.056 (3)	
C3	0.5091 (8)	0.8444 (5)	0.500 (2)	0.066 (3)	
H3	0.4330	0.8573	0.4098	0.079*	
C4	0.5267 (8)	0.7679 (5)	0.646 (3)	0.070 (3)	
H4	0.4617	0.7294	0.6501	0.084*	
C5	0.6369 (8)	0.7465 (4)	0.787 (2)	0.060 (3)	
C6	0.7312 (8)	0.8044 (5)	0.779 (2)	0.058 (3)	
H6	0.8056	0.7925	0.8792	0.069*	
C7	0.6509 (9)	0.6621 (5)	0.946 (2)	0.090 (3)	
H7A	0.7023	0.6684	1.1261	0.135*	
H7B	0.6886	0.6226	0.8070	0.135*	
H7C	0.5706	0.6417	1.0072	0.135*	
C8	0.5000	1.0000	0.168 (4)	0.080 (5)	
H8A	0.4805	0.9524	0.0361	0.096*	0.50
H8B	0.5195	1.0476	0.0361	0.096*	0.50

### Atomic displacement parameters (Å^2^)

**Table:**

	*U*^11^	*U*^22^	*U*^33^	*U*^12^	*U*^13^	*U*^23^
Br1	0.0546 (6)	0.0668 (6)	0.1623 (14)	−0.0129 (5)	−0.0131 (8)	0.0130 (8)
O1	0.049 (3)	0.062 (3)	0.089 (5)	0.017 (3)	0.004 (4)	0.012 (4)
C1	0.052 (4)	0.042 (4)	0.066 (7)	0.009 (3)	0.001 (5)	0.004 (5)
C2	0.048 (4)	0.056 (5)	0.062 (8)	0.020 (4)	0.010 (5)	−0.001 (5)
C3	0.043 (4)	0.081 (6)	0.074 (8)	0.006 (5)	−0.009 (5)	−0.012 (6)
C4	0.058 (5)	0.061 (5)	0.090 (9)	−0.011 (4)	0.009 (7)	−0.008 (7)
C5	0.064 (5)	0.041 (4)	0.074 (8)	−0.006 (4)	0.020 (5)	−0.001 (5)
C6	0.058 (5)	0.057 (5)	0.058 (7)	0.008 (4)	0.007 (5)	0.002 (5)
C7	0.110 (8)	0.062 (5)	0.098 (9)	−0.002 (6)	0.019 (9)	0.026 (6)
C8	0.082 (10)	0.077 (9)	0.081 (12)	0.023 (8)	0.000	0.000

### Geometric parameters (Å, °)

**Table:**

Br1—C1	1.882 (7)	C5—C6	1.370 (10)
O1—C2	1.364 (9)	C5—C7	1.515 (10)
O1—C8	1.412 (10)	C6—H6	0.9300
C1—C2	1.363 (11)	C7—H7A	0.9600
C1—C6	1.385 (10)	C7—H7B	0.9600
C2—C3	1.417 (11)	C7—H7C	0.9600
C3—C4	1.383 (11)	C8—O1^i^	1.412 (10)
C3—H3	0.9300	C8—H8A	0.9700
C4—C5	1.376 (12)	C8—H8B	0.9700
C4—H4	0.9300		
			
C2—O1—C8	118.0 (6)	C5—C6—C1	121.0 (8)
C2—C1—C6	122.1 (7)	C5—C6—H6	119.5
C2—C1—Br1	119.4 (6)	C1—C6—H6	119.5
C6—C1—Br1	118.4 (6)	C5—C7—H7A	109.5
C1—C2—O1	117.0 (7)	C5—C7—H7B	109.5
C1—C2—C3	117.6 (8)	H7A—C7—H7B	109.5
O1—C2—C3	125.5 (8)	C5—C7—H7C	109.5
C4—C3—C2	119.2 (8)	H7A—C7—H7C	109.5
C4—C3—H3	120.4	H7B—C7—H7C	109.5
C2—C3—H3	120.4	O1—C8—O1^i^	111.2 (12)
C5—C4—C3	122.5 (8)	O1—C8—H8A	109.4
C5—C4—H4	118.7	O1^i^—C8—H8A	109.4
C3—C4—H4	118.7	O1—C8—H8B	109.4
C6—C5—C4	117.6 (8)	O1^i^—C8—H8B	109.4
C6—C5—C7	122.0 (9)	H8A—C8—H8B	108.0
C4—C5—C7	120.3 (8)		
			
C6—C1—C2—O1	177.7 (8)	C2—C3—C4—C5	1.0 (16)
Br1—C1—C2—O1	1.3 (13)	C3—C4—C5—C6	0.2 (16)
C6—C1—C2—C3	−2.4 (15)	C3—C4—C5—C7	179.6 (9)
Br1—C1—C2—C3	−178.8 (7)	C4—C5—C6—C1	−2.5 (15)
C8—O1—C2—C1	170.0 (9)	C7—C5—C6—C1	178.1 (8)
C8—O1—C2—C3	−9.8 (14)	C2—C1—C6—C5	3.7 (15)
C1—C2—C3—C4	0.1 (15)	Br1—C1—C6—C5	−179.8 (7)
O1—C2—C3—C4	179.9 (9)	C2—O1—C8—O1^i^	76.7 (6)

Symmetry codes: (i) −*x*+1, −*y*+2, *z*.
